# Rehabilitation of Neuromuscular Diseases During COVID-19: Pitfalls and Opportunities

**DOI:** 10.3389/fneur.2021.626319

**Published:** 2021-02-19

**Authors:** Sara Liguori, Antimo Moretti, Marco Paoletta, Francesca Gimigliano, Giovanni Iolascon

**Affiliations:** ^1^Department of Medical and Surgical Specialties and Dentistry, University of Campania “Luigi Vanvitelli”, Naples, Italy; ^2^Department of Mental and Physical Health and Preventive Medicine, University of Campania “Luigi Vanvitelli”, Naples, Italy

**Keywords:** COVID-19, neuromuscular diseases, respiration disorders, muscle wasting, rehabilitation, home-based program

## Abstract

The outbreak of COVID-19 caused by SARS-CoV-2 has spread worldwide with a huge impact on the healthcare system. Compared to the previous coronaviruses-related pandemics, COVID-19 is more transmissible with potential systemic involvement and peculiar neurological manifestations, such as Guillan-Barrè syndrome up to critical illness myopathy, occurring in the intensive care setting. In this clinical scenario, people living with a neuromuscular disease (NMD) represent a vulnerable category with a high risk of a severe course of COVID-19. Moreover, in the NMD population, the management of respiratory and muscular impairments after SARS-CoV-2 infection might be troubling in terms of both pharmacological and rehabilitative approaches. To date, rehabilitation is still an unmet need in this population with several implications on NMD progression with and without SARS-CoV-2 infection. In particular, rehabilitation intervention for patients with NMD after COVID-19 are lacking. Therefore, in the current paper, we analyze the critical issues of COVID-19 on NMDs patients and propose a home-based rehabilitation program targeted for this population after mild to moderate SARS-CoV-2 infection.

## Introduction

The COVID-19 pandemic caused by SARS-CoV-2 continues to act as a great burden for social and healthcare systems in the modern era due to unexpected contagiousness and severity of the clinical condition (1–3). Similar to other CoVs (SARS-CoV and Middle East respiratory syndrome coronavirus, MERS-CoV), SARS-CoV-2 appears to be neurotropic since peculiar manifestations (dysgeusia, anosmia, seizures, and even encephalitis) might occur in patients affected by COVID-19 (4–6).

Neurological involvement can be equally due to post-infectious immune-mediated mechanisms as in Guillan-Barrè syndrome (GBS) (7, 8) or myositis (9). Moreover, neuromuscular complications of COVID-19 such as critical illness myopathy (CIM), polyneuropathy (CIP), and polyneuromyopathy (CIPNM) might occur during intensive care stay (10).

Patients affected by neuromuscular diseases (NMDs) can experience more frequent and severe COVID-19-related complications compared to the general population (11, 12).

NMDs are rare conditions (prevalence < 1/2,000) involving anterior horn cells, peripheral nerves, neuromuscular junctions, or skeletal muscles that result in poor motor performance (13). Management of NMD patients affected by COVID-19 is challenging and requires specific pharmacological and rehabilitation strategies even after the resolution of respiratory infection. The aim of this paper is to analyze the critical issues of COVID-19 on NMDs patients and to propose a home-based rehabilitation program for this population after SARS-CoV-2 infection.

## COVID-19-Related Respiratory and Muscle Impairments in NMDs

### COVID-19 and Respiratory Involvement in NMDs

In patients affected by NMDs, respiratory impairment is common and includes inspiratory and expiratory muscle weakness, ineffective cough, alteration of blood gases, nocturnal sleep disorder, reduction of vital capacity, and dyspnea during activities of daily living (ADLs), strongly contributing to progressive disability (14, 15). Therefore, this population is at higher risk of recurrent lower respiratory tract infections and acute respiratory failure that increase hospitalization and mortality rates (16).

To better define the impact of COVID-19 on NMDs patients with respiratory involvement, several issues should be examined. Some pre-existing impairments might contribute to the occurrence of COVID-19 complications in NMDs, such as a force vital capacity (FVC) of <60%, use of ventilation, or swallowing dysfunctions (Table 1) (12). Moreover, spine deformities, such as kyphoscoliosis, commonly observed in these patients, might further worsen respiratory function by reducing chest expansion, thus contributing to an increased risk of developing respiratory distress during COVID-19 (17, 18). On the other hand, the mildest forms of NMD with stabilized cardiopulmonary function without other comorbidities seem to be associated with lower rates of COVID-19 complications and better respiratory outcomes, even after hospitalization in intensive care (18).

**Table 1:** Risk factors of severe COVID-19 course in NMDs patients according to the World Muscle Society (WMS) (11).

Muscular weakness of the chest or diaphragm, resulting in respiratory volumes <60% predicted (FVC < 60%), especially in patients with kyphoscoliosis
Use of ventilation via mask or tracheotomy
Weak cough and weak airway clearance due to oropharyngeal weakness
Presence of tracheostoma
Cardiac involvement (and/or on medication for heart involvement)
Conditions at risk of deterioration with fever, fasting, or infection (e.g., neuromuscular junction or metabolic disorders)
Conditions at risk of rhabdomyolysis with fever, fasting, or infection
Comorbidity (e.g., diabetes and obesity)
Patients receiving steroids and immunosuppressant treatment

However, the coexistence of NMDs and COVID-19 is a dangerous duet to be managed, particularly from a rehabilitative perspective. For example, in case of interstitial pneumonia due to SARS-CoV-2 infection, supplemental oxygen therapy should be provided to avoid hypoxemia, but if this intervention is not combined with adequate ventilatory support, worsening of underlying chronic hypercapnia in NMDs patients might occur because of rapid onset of respiratory muscles exhaustion (19, 20). Otherwise, for some NMDs characterized by severe bulbar involvement, the use of ventilation devices might be poorly tolerated (21). In the case of acute respiratory distress syndrome (ARDS), endotracheal intubation is required and prone positioning is often preferred (22), but NMDs patients might be affected by limited neck mobility and/or oropharyngeal muscle atrophy so that these procedures might cause severe discomfort (23, 24).

For post-acute COVID-19 patients affected by NMDs, it is necessary to identify the signs of residual pulmonary damage, such as ground-glass opacities, consolidation, pleural effusion, and irregular solid nodules, and their implications on functioning to design a patient-tailored rehabilitation approach (25).

### COVID-19 and Muscular Involvement in NMDs

At the tissue level, patients with NMDs have skeletal muscles, motor nerves, or neuromuscular junctions' damages with progressive loss of functional ability and poor perceived quality of life (13). For this population, COVID-19 represents a precipitating factor of muscle wasting, also through the deconditioning due to inactivity and the high risk of long-term bedridden.

Several factors might contribute to poor functional recovery in NMD patients affected by COVID-19 (9). During SARS-CoV-2 infection, myalgia is a frequent complaint with a prevalence ranging from 21.9 to 35.8% of COVID-19 patients (26). Pitscheider et al. have observed that muscle damage seems to occur in a similar frequency in COVID-19 and influenza infection but with higher severity in influenza, as suggested by significantly higher serum creatine kinase (CK) levels compared to COVID-19. Authors claimed that it is difficult to determine whether this finding is due to a virus-triggered inflammatory response or to direct muscle toxicity (27). It should be underlined that have been described episodes of rhabdomyolysis associated with autoimmune COVID-19-related myositis, up to the CIM (28, 29). Furthermore, as a consequence of the chronic inflammatory process, people affected by COVID-19 often develop muscle wasting with a poor physical performance that leads to mobility limitation contributing to a loss of independence and increased hospitalization and healthcare cost (30).

This infection might exacerbate the clinical and functional complaints of NMD patients. For example, Anand et al. described five cases of COVID-19-related worsening of myasthenia gravis successfully treated with intravenous immunoglobulins and steroid administration (31).

On the contrary, the pattern and severity of muscle involvement in NMD (e.g., reversible, slow, and rapidly progressing NMDs) should be carefully considered as potential prognostic factors for COVID-19 in terms of rehabilitation outcomes since progressive muscle wasting, joint contractures, and fatigue characterize NMD patients (32).

## Practical Considerations for NMD Patients During COVID-19

### Management of Pharmacological Therapy

During the COVID-19 era, for people with NMDs is not recommended to discontinue ongoing treatments; for example, in those affected by Duchenne or Becker Muscular Dystrophy (DBMD), steroid cessation is not advised and eventually adjusted in case of COVID-19 to avoid adrenal insufficiency (19). Moreover, in a recent study about drugs identified as “protective” against this infectious disease, it has been demonstrated that the administration of ubiquinone, a supplement commonly used in DBMD patients, might reduce the risk of COVID-19-related hospitalization (33).

Similarly, in the inflammatory myopathies, myasthenia gravis, and peripheral nerve disease, previous administration of immunosuppressive drugs, like azathioprine or methotrexate, should not be discontinued, except in selected cases, under the supervision of neuromuscular specialists (34). Nevertheless, some pharmacological treatments that need hospitalization, such as eteplirsen and other exon skipping for DBMD and enzyme replacement therapies (ERT) like recombinant human GAA (rhGAA) in Pompe Disease (PD), have stopped during a pandemic due to the risk for these patients to contract an infection while in hospital (19). For the PD population, there are some data reporting no change in the disease course after ERT discontinuation for <3 months, while a severe deterioration of muscular and respiratory functions might occur if ERT is discontinued for over 9 months (35). An alternative option is a home infusion to minimize the exposure to COVID-19 (19). On the other hand, for spinal muscular atrophy (SMA), the administration of intrathecal nusinersen is mandatory, despite the COVID-19 pandemic, due to the proven worsening of clinical and functional condition with delayed treatment in particular for SMA 1 and young children with SMA 2, while for the adolescent or adults, injections could be delayed for a maximum of 4 months from the last administration (36, 37).

For what concern drugs commonly used to treat COVID-19 patients, a lack of reliable evidence persists (38).

In Table 2 are reported antiviral and immunomodulatory drugs currently under evaluation according to the COVID-19 Treatment Guidelines Panel (38), their mechanism of actions, route of administration, and safety issues in NMDs patients (39, 40).

**Table 2:** Mechanism of action, route of administration, and safety of drugs used in clinical practice for COVID-19 therapy.

**Drugs**	**Route of administration**	**Mechanism of action**	**Safety issues in NMD patients**
Chloroquine phosphate Hydroxychloroquine	Oral	Interfere with entry of the virus into human cells and inhibition of virus replication Increase the PH of endosomes and lysosomes, inactivating of t-cells and other cytokines Interfere with antigen presentation, decreasing the antigen–major histocompatibility complex (MHC)	Prolonged QT interval, Torsades de Pointes, Atrioventricular block, ventricular arrhythmia; neuromyopathy and myopathy
Lopinavir/Ritonavir	Oral	Inhibit the action of the enzyme 3-chymotrypsin-like protease (3-clpro) disrupting the process of viral replication and release from host cells	PR or QT prolongation
Remdesivir	Intravenous	Inhibit the viral RNA synthesis binding the viral RNA-dependent RNA polymerase (RDRP) complexes	Mild, reversible PT prolongation
Tocilizumab	Intravenous	Inhibit IL-6 receptor, blocks cytokine storm	Risk of serious opportunistic infections
Dexamethasone	Oral or Intravenous	Modulate inflammation-mediated lung injury	Risk of serious opportunistic infections
Azithromycin	Oral	Decrease the virus entry into cells Up-regulate genes involved in the innate response	QT prolongation

Among the adjunctive therapies, a potential role in the treatment of COVID-19 has been proposed for two vitamins: vitamin C as an antioxidant and free radical scavenger and vitamin D as a modulator of innate and adaptive immune responses (41, 42). However, the lack of compelling data requires further study to make reliable recommendations on the use of these vitamins during COVID-19 (38).

### Management of Rehabilitation Needs

Rehabilitation is an unmet need in the COVID-19 era, particularly for NMDs patients. During the COVID-19 pandemic, these patients experienced serious difficulties to receive usual rehabilitation care with potential implications on disease progression as well as functional worsening and psychological distress (32); moreover, considering several respiratory and musculoskeletal sequelae, including mild cases in home-isolation, COVID-19 might significantly worsen functional outcomes achieved through rehabilitative approach in these patients (32).

According to a recent expert consensus on rehabilitation protocol for COVID-19 patients, it was proposed a comprehensive evaluation and rehabilitation intervention based on the International Classification of Functioning, Disability and Health (ICF), using ICF core set for chronic obstructive pulmonary disease (COPD) (43). Nevertheless, no systematic identification of impairment and disability according to the ICF was made for NMD patients after COVID-19. In this context, only care guidance and one expert opinion address the specific needs in this population, such as pharmacological management, the role of telemedicine, and emergency procedures, to adopt (19, 32). Regarding rehabilitative approaches to NMDs patients after COVID-19, the main issues are the lack of dedicated protocols, including measurement tools and functional outcomes to achieve, and insufficient data about the safety of rehabilitation procedures. Although telemedicine or home-based rehabilitation are potential resources to provide care for these patients, evidence supporting their use is scant (17).

In the current paper, we propose a home-based rehabilitation program for the management of respiratory and muscle impairment in NMD patients after COVID-19.

## A Proposal for Home-Based Rehabilitation for NMDs Patients After SARS-CoV-2 Infection

According to the available recommendations on physiotherapy for NMDs and the respiratory and muscle impairment after COVID-19 (44–49), we designed a home-based rehabilitation program, intended for ambulatory patients affected by genetically confirmed NMDs after asymptomatic, mild, or moderate COVID-19 (i.e., at discharge from hospital or testing negative for the virus, excluding those requiring non-invasive ventilation or tracheostomy).

Our proposed approach aims to preserve and/or progressively increase respiratory muscle strength, optimize endurance and exercise tolerance, reducing fatigue in this population. Our protocol include a preliminary evaluation, useful to suggest the most appropriate rehabilitation program, consisting of the following assessments: collection of demographic and anthropometric data; vital signs measurement (body temperature, respiratory and heart rate, blood pressure, blood oxygen saturation); spirometry to measure the maximal inspiratory pressure (MIP) and maximal expiratory pressure (MEP), which are useful to assess the strength of respiratory muscles; evaluation of all three dimensions of motor performance (standing and transfer, axial and proximal motor function, and distal motor function) through the Motor Function Measure-32 (MFM-32) (50); timed test as the Six-Minute Walk Test (6 MWT) or Two-Minute Walk Test (2 MWT) to investigate aerobic capacity and endurance (51, 52); evaluation of fatigue by Fatigue Severity Scale (FSS) (53); assessment of perceived Quality of Life (QoL) through the EuroQoL five-dimensions (EQ-5D) (54); characterization of functioning and disability using ICF GENERIC-30 SET; and functional limitation due to respiratory and motor impairment through the Barthel Index Dyspnea (55) (Table 3). During the evaluation step, the examiner will administer the Borg Category-Ratio (CR-10) Scale pre- and post 6 MWT, together with monitoring of vital signs, to measure physical activity intensity. Moreover, Borg CR-10 will be explained to the patient for self-administration during home training, aimed to guide the exercise intensity. After these preliminary assessments, it will provide an exercise educational program describing the tailored home-based rehabilitation, according to patients' functioning and motivation, and defining appropriate exercise parameters, using the FITT principle (i.e., frequency, intensity, time, and type). Our proposed intervention is principally focused on Respiratory Muscle Training (RMT), Aerobic Reconditioning, Resistance Training, and daily sessions of Lung Recruitment Techniques.

**Table 3:** Our proposed evaluation protocol for people affected by NMDs post COVID-19.

Demographic and anthropometric data
Vital signs
Spirometry
ICF GENERIC-30 SET
Motor Function Measure-32 (MFM-32)
Six-Minute Walk Test (6 MWT) or Two-Minute Walk Test (2 MWT)
Fatigue Severity Scale (FSS)
EuroQoL five-dimensions (EQ-5D)
Barthel Index Dyspnea
Borg Category-Ratio Scale

A physiotherapist will provide theoretical instructions for each exercise together with video demonstrations available online on specific web-based platforms.

In detail, the RMT program consists of respiratory muscle strengthening using a pressure threshold loading, to increase both MIP and endurance of inspiratory muscles, with a suggested frequency of at least 3 × 30 min/week and an intensity of 30–70% MIP/MEP. Aerobic Reconditioning initially provides march on spot and step-ups and progressively introduces free walking to then move on cycling; also in this case the frequency suggested is 3 × 30 min/week, with an intensity below anaerobic threshold, established by the onset of dyspnea/fatigue symptoms or a Borg CR-10 score below 3. Resistance Training includes both open-kinetic-chain and closed-kinetic-chain exercises for upper and lower limbs for 20 min 2–3 times/week and low-intensity effort (60% of effort of one repetition maximum, 1 RM), where the one repetition maximum indicates the maximum weight that a person can lift in a single repetition. Lung Recruitment Techniques include controlled breathing (deep-slow breathing using diaphragm to improve ventilation/perfusion value), paced breathing (with respect to the effort), chest expansion breathing combined with shoulders retraction to increase respiratory compliance, active cycle of breathing techniques, and a Positive Expiratory Pressure (PEP) trainer to facilitate airway clearance and improve lung function.

Moreover, patients may register their daily number of steps through a common smartphone pedometer application to monitor their walking performance. It would be appropriate for the patient to be closely monitored (at least every month from the beginning of the training). The home-based rehabilitation program proposed being reported in Table 4.

**Table 4:** Proposal of home-based rehabilitation program for NMDs patients after COVID-19.

**Prescription**	**Frequency**	**Intensity**	**Time**	**Type**
RMT strength	3 sessions × 30 min a week	30–70% MIP/MEP	At least 8–10 weeks	Pressure threshold loading
Aerobic reconditioning	3 sessions × 30 min a week	Below anaerobic threshold^*^	At least 8–10 weeks	March on spot, step-ups, free walking, cycling
Resistance training	2–3 sessions × 20 min a week	60% of 1RM	At least 8–10 weeks	OKC and CKC exercises for upper and lower limb
Lung recruitment tecniques	5–10 min × max. 3 times a day	At vital capacity level or at 3 of Borg CR-10 scale	At least 8–10 weeks	Controlled breathing; paced breathing; Chest expansion breathing; Active cycle of breathing techniques; PEP trainer

* *Established by onset of dyspnea/fatigue symptoms or a score below 3 of Borg CR-10 scale*.

*RMT, Respiratory Muscle Training; MIP, maximal inspiratory pressure; MEP, maximal expiratory pressure; RM, repetition maximum; OKC, open-kinetic-chain; CKC, closed-kinetic-chain; Borg CR-10, Borg Category-Ratio Scale; PEP, Positive Expiratory Pressure*.

## Conclusion

During the COVID-19 era, several critical issues in the NMD population have been reported. These patients experience changes in the management of ongoing drug treatment or may discontinue rehabilitative care. Moreover, in NMD patients, a severe course of COVID-19 might occur, with uncertain prognosis and complications often requiring intensive care stay. On the other hand, some NMD patients appear in a mild to moderate clinical course of COVID-19, with a better perspective of both respiratory and muscle recovery. In this clinical scenario, rehabilitation is still an unmet need, with a lack of operational protocol that is specific for this population. Our home-based rehabilitation program proposal is intended to preserve and/or improve respiratory muscle strength, endurance and exercise tolerance, counteracting fatigue in NMD patients after COVID-19.

## Data Availability Statement

The original contributions presented in the study are included in the article/supplementary material, further inquiries can be directed to the corresponding author/s.

## Author Contributions

GI and SL contributed to the conception and design of the paper. SL and AM wrote the first draft of the manuscript. All authors contributed to manuscript revision and read and approved the submitted version.

## Conflict of Interest

The authors declare that the research was conducted in the absence of any commercial or financial relationships that could be construed as a potential conflict of interest.
